# Characterizing Mobilized Virulence Factors and Multidrug Resistance Genes in Carbapenemase-Producing *Klebsiella pneumoniae* in a Sri Lankan Hospital

**DOI:** 10.3389/fmicb.2018.02044

**Published:** 2018-08-31

**Authors:** Chendi Zhu, Veranja Liyanapathirana, Carmen Li, Vasanthi Pinto, Mamie Hui, Norman Lo, Kam T. Wong, Nilanthi Dissanayake, Margaret Ip

**Affiliations:** ^1^Department of Microbiology, The Chinese University of Hong Kong, Shatin, Hong Kong; ^2^Department of Microbiology, Faculty of Medicine, University of Peradeniya, Kandy, Sri Lanka; ^3^Department of Anesthesiology and Critical Care, Faculty of Medicine, University of Peradeniya, Kandy, Sri Lanka

**Keywords:** OXA-181, quinolone, yersiniabactin, *Klebsiella pneumoniae*, Sri Lanka

## Abstract

Limited data is available on the epidemiology and characteristics of carbapenem-resistant *Enterobacteriaceae* (CRE) and their associated plasmids or virulence determinants from Sri Lanka. Through whole genome sequencing of CREs from the intensive care units of a Sri Lankan teaching hospital, we identified a carbapenemase gene, *bla*_OXA–181_ in 10 carbapenemase-producing *Klebsiella pneumoniae* isolates (two strains of ST437 and eight strains of ST147) from 379 respiratory specimens. *bla*_OXA–181_ was carried in three variants of ColE-type plasmids. *K. pneumoniae* strains with *ompK36* variants showed high minimum inhibitory concentrations to carbapenem. Furthermore, genes encoding for extended spectrum β-lactamases (ESBL), plasmid-mediated quinolone resistance (PMQR) determinants (*qnr*, *aac(6′)-Ib-cr*, and *oqxAB*) were present in all 10 strains. Amino acid substitution in chromosomal quinolone resistance-determining regions (QRDRs) *gyrA* (Ser83Ile) and *parC* (Ser80Ile) were also observed. All strains had yersiniabactin genes on mobile element ICE*kp*. Strict infection control practices and judicious use of antibiotics are warranted to prevent further spread of multidrug-resistant *K. pneumoniae*.

## Introduction

Carbapenem-resistant *Enterobacteriaceae* (CRE) is a global threat and infections caused by CRE are associated with high morbidity and mortality (Centers for Disease Control and Prevention, 2013). Among all the resistance mechanisms, plasmid-mediated horizontal transfer of carbapenemase genes is the main route for acquiring resistance in CRE (Nordmann et al., 2012). These mobile elements are capable of transferring resistance between different lineages and thus, pose a potential for dissemination. Three types of carbapenemases (class A: *bla*_KPC_; class B: *bla*_NDM_, *bla*_IMP_, and *bla*_V IM_; class D: *bla*_OXA–48–like_) can hydrolyze carbapenems at varying levels along with other mechanisms, such as porin mutation and overexpression of efflux pump proteins (Poirel et al., 2012; Zhang et al., 2014; Lunha et al., 2016). *Klebsiella pneumoniae* clonal complex 258 (CC258) have been known to associate with epidemic plasmids carrying numerous antimicrobial resistance genes and virulence factors like yersiniabactin. These interactions were hypothesized to provide a survival advantage for these clones (Holt et al., 2015). Sri Lanka sits in the southern tip of the Indian subcontinent. Although the epidemiology and diversity of carbapenemases, have been reported in India, the epidemiology and characteristics of the CRE and resistance determinants in Sri Lanka is lacking. Furthermore, the single study currently available on CRE from Sri Lanka does not contain information on associated plasmids or virulence determinants (Hall et al., 2014). Hence, we sequenced the CRE strains isolated from patient specimens in the intensive care units of one Sri Lankan hospital using whole genome sequencing (WGS) to describe their antimicrobial resistance and genetic profiles so as to provide a more in-depth view of the CRE in Sri Lanka.

## Materials and Methods

### Bacterial Isolates

Single patient isolates were obtained from the respiratory specimens received from inpatients admitted to the intensive care units of the Teaching Hospital, Peradeniya, Sri Lanka between February to September 2015. During this period, a total of 379 respiratory specimens were processed and 64 coliforms were obtained. Among these coliforms, 2.6% (10 isolates) were found to be resistant to carbapenems using Stokes sensitivity testing method (Sri Lanka College of Microbiologists, 2011) and were saved for further study. The study was approved by the Institutional Ethical Committee of the Faculty of Medicine, University of Peradeniya.

### Bacterial Identification and Antimicrobial Resistance

Bacterial strains were plated on blood agar and incubated at 37°C overnight and the identification of all strains were confirmed by matrix-assisted laser desorption ionization-time of flight (MALDI-TOF) mass spectrometry at the Department of Microbiology, the Chinese University of Hong Kong. Minimum inhibitory concentrations (MICs) of antimicrobials were determined by the microbroth dilution method according to Clinical and Laboratory Standards Institute (CLSI) guideline (CLSI, 2018). The following antibiotics were tested: amikacin (AK); ceftazidime (CAZ); ciprofloxacin (CIP); gentamicin (GN); colistin (CT); cefotaxime (CTX); ertapenem (ETP); fosfomycin (FOS); imipenem (IPM); meropenem (MEM); and tigecycline (TG).

### Whole Genome Sequencing and Data Analysis

Bacterial DNA was extracted with Wizard genomic DNA purification kit (Promega, Madison, WI, United States). WGS was performed using the Illumina HiSeq 2500 platform, and unique index-tagged libraries were created for each sample to generate 90 bp paired-end reads (Global Biologics, LLC). The libraries gave 100× average coverage for each strain. Quality control of the raw reads was performed by FastQC (Andrews, 2010). Genomes were assembled using SPAdes assembler (version 3.5.0) (Bankevich et al., 2012). Contigs of ≥500 bp from each genome were included in the analyses. Prokka (version 1.9) software was used for genome annotation, including ORF finding and gene function annotation (Seemann, 2014). Raw reads and assembled contigs were used for multilocus sequence typing (MLST) analysis. SRST2 (Version 0.1.5) was used to map raw reads to the following databases: pubMLST database for sequence types; ARG-ANNOT V3 database for resistance genes; PlasmidFinder database (Updated 20170220) for plasmid replicons; and *K. pneumoniae* BIGSdb virulence gene database for virulence genes at http://bigsdb.web.pasteur.fr (Accession date: 20180313) (Carattoli et al., 2014; Gupta et al., 2014; Inouye et al., 2014). The contig containing carbapenemase for each genome was matched against the public database with NCBI BLAST to find the top hit plasmids of at least 95% identity and 95% coverage and were matched back to the plasmids in our genome dataset to extract possible plasmid contigs (Altschul et al., 1990). Extracted contigs were further aligned to the reference plasmid using Mauve Alignment software to check for similarity and coverage (Darling et al., 2004). Gaps were filled by PCR with primers designed from our genome data. Pan-genome dendrogram was created by Roary (Page et al., 2015). Contig files of all 10 strains in this study were deposited to GenBank BioProject under the accession number PRJNA439172.

## Results

### Antimicrobial Susceptibilities and Molecular Characteristics of the CRE Isolates

Of the 10 CRE isolates included in the study, three were from the subsidiary ICU and seven were from the main ICU. All strains were confirmed *K. pneumoniae* and the susceptibility testing revealed all strains to be resistant to CIP, CTX, and sensitive to FOS and AK, while MIC for CT was ≤0.5 mg/L and MIC for TG was ≤2 mg/L (**Table 1**). All except one strain (SL50) were resistant to CAZ. All of the strains except SL33, SL49, and SL54 were resistant to ETP or MEM with MICs ≥16 and ≥8 mg/L, respectively, while resistance to IPM varied among isolates. In previous studies, mutations of *K. pneumoniae* outer membrane proteins ompK35 and ompK36 were found to confer increased MIC to carbapenems (Garcia-Fernandez et al., 2010; Zhang et al., 2014; Lunha et al., 2016). There were neither mutations nor insertions at ompK35 and its promoter in all of our strains when we aligned them to the wild-type gene in public database (GenBank Accession No. AJ011501). OmpK36 was identical in six highly resistant strains (ompK36-sl1). SL33 and SL49 shared the same gene (ompK36-sl2), but SL54 contained a unique variation (ompK36-sl3). The difference of ompK36-sl1 and ompK36-sl2 was the insertion of amino acids glycine and aspartate after PEFXG domain within the L3 loop and one mutation in loop L4 and alpha-helix, respectively. The PEFXG domain (porin size determinant) insertion was also observed in several studies that addressed strains with high resistance to carbapenems (Garcia-Fernandez et al., 2010; Hall et al., 2014; Zhang et al., 2014). OmpK36-sl3 was different from the others in the region between loop L3 and loop L6, but there was no insertion interruption in the PEFXG domain.

**Table 1:** Characteristics of the *bla*_OXA–181_ producing *K. pneumoniae* isolates.

Strain No	Unit^∗∗^	Stay length^∗∗∗^ (days)	Specimen	ST	CUHK_SL	Otherplasmidsreplicons	*ompK*36 PEFXG insertion	MICs(mg/L)^∗^										
								ETP	IPM	MEM	CTX	CAZ	AK	GN	CIP	TG	CT	FOS
SL33	S	–	ET secretion	437	A	IncFIB, IncFII	No	2	0.25	0.25	>128	64	2	64	64	1	0.12	4
SL49	M	7	ET secretion	437	A	IncFIB	No	4	0.5	0.25	128	128	4	0.5	128	1	0.25	4
SL54	S	–	ET secretion	147	C	IncFIB, IncR	No	4	1	0.5	>128	64	4	64	128	2	0.12	8
SL34	M	4	Sputum	147	B	IncFIB, IncA/C2, IncR	Yes	32	4	32	>128	>128	4	64	128	0.5	0.5	8
SL35	M	6	Sputum	147	B	IncFIB, IncR	Yes	32	4	32	>128	>128	4	128	64	0.5	0.25	16
SL36	M	17	Sputum	147	B	IncFIB, IncFII	Yes	32	8	32	>128	32	2	1	8	1	0.12	16
SL46	M	9	ET secretion	147	B	IncFIB, IncFII	Yes	16	1	8	128	16	4	0.5	8	1	0.12	8
SL50	M	13	ET secretion	147	B	IncFIB, IncR	Yes	32	8	16	4	0.5	1	128	32	1	0.25	32
SL52	S	–	ET secretion	147	B	IncFIB, IncR	Yes	32	2	32	>128	>128	4	128	16	1	0.25	64
SL68	M	4	Sputum	147	B	IncFIB, IncR	Yes	32	8	32	>128	>128	16	1	8	1	0.25	64


*^∗^AK, amikacin; CAZ, ceftazidime; CIP, ciprofloxacin; GN, gentamicin; CT, colistin; CTX, cefotaxime; ETP, ertapenem; FOS, fosfomycin; IPM, imipenem; MEM, meropenem; TG, tigecycline.*

*^∗∗^M – Main study unit; S – Subsidiary unit.*

*^∗∗∗^Length of stay from date of specimen collection.*

**FIGURE 1:**
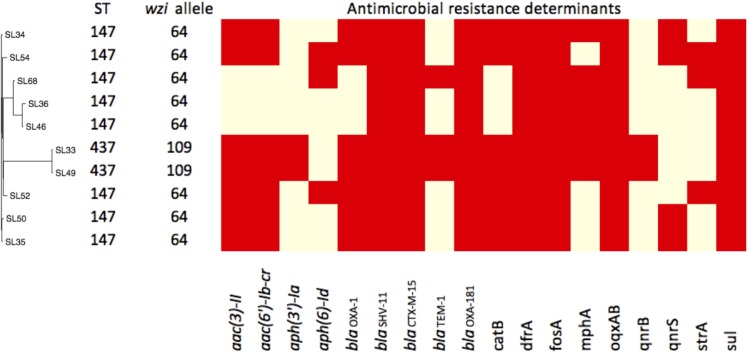
Pan-genome dendrogram of 10 Sri Lanka strains with annotation. ^∗^Red boxes indicate presence and yellow for absence.

The *K. pneumoniae* isolates belonged to ST437 (2 strains) and ST147 (8 strains) (**Table 1**). Capsular genes showed two *wzi* alleles 64 and 109 in strains ST147 and ST437, respectively (**Figure 1**). Only one carbapenemase gene, *bla*_OXA–181_ was found in all isolates. All isolates harbored β-lactamase genes, including *bla*_TEM-1_, and *bla*_OXA-1_, *bla*_SHV -11_, and to extended-β-lactamase, *bla*_CTX-M-15_. Plasmid-mediated quinolone resistance (PMQR) determinants (*qnr*, *aac(6′)-Ib-cr*, and *oqxAB*) were also detected. We also found two amino acid substitutions at *gyrA* (S83I) and *parC* (S80I) in all strains, which have been frequently reported in quinolone-resistant *K. pneumoniae* worldwide (Aldred et al., 2014).

The virulence profiles among the 10 strains were the same and all carried yersiniabactin genes (*ybt*, *irp1*, *irp2*, and *fuyA*) and *kfu*, *mrk*, but were absent of *rmpA* or *rmpA2* genes. Yersiniabactin genes were found on mobile element ICE*kp*. 4437 genes and 2182 accessory genes were used to establish the dendrogram of their pan-genomes (**Figure 1**). The distinct accessory gene profiles of ST437 and ST147 indicated that ST437 was a distant cluster. The pan-genome dendrogram also revealed a cluster of ST147 (SL36, SL46, and SL68) that contained fewer resistance genes (**Figure 1**) and were sensitive to GN and with lower MIC (8 mg/L) to CIP compared with other ST147 strains (**Table 1**).

### Plasmids Harboring *bla*_OXA–181_

**FIGURE 2:**
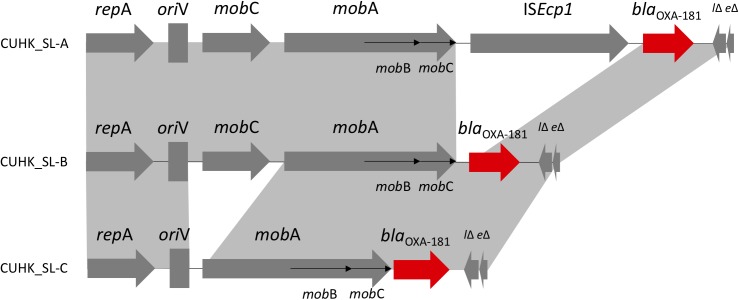
ColE-type plasmids in Sri Lanka. ^∗^CUHK_SL-B had IS*Ecp1* deletion; CUHK_SL-C had IS*Ecp1* and *mob*C deletion.

Three different ColE-type plasmids were identified (**Figure 2**). One plasmid (CUHK_SL-A) was identical to KP3 (GenBank accession no. JN205800) and was found in the two ST437 strains. CUHK_SL-A was a short plasmid (7,606 bp) harboring only one resistance gene and was previously well described (Potron et al., 2011a). Another plasmid (CUHK_SL-B) was found in 7 ST147 strains of this study with a deletion of an insertion sequence (IS*Ecp1*) when compared to CUHK_SL-A. Several studies from the United States, Germany, and France have reported this plasmid in both *K. pneumoniae* and *Escherichia coli* (GenBank Accession No. CP006802, CP016038, and JX423831). The third plasmid (CUHK_SL-C) was found in strain SL54 with a ^∗∗^mobile gene deletion (*mobC*) when compared to CUHK_SL-B. This plasmid was not reported before.

## Discussion

Carbapenemase *bla*_OXA–181_ was first described in India as a Class D *bla*_OXA–48–like_ enzyme from clinical samples in year 2006 and 2007 (Castanheira et al., 2011). It was thought to originate from an environmental strain as a chromosomal gene (Potron et al., 2011b). Although it has been detected worldwide, most of the patients have a travel history to the Indian subcontinent, especially India (Poirel et al., 2012). In 2014, *bla*_OXA–181_ and *bla*_NDM_ were reported in *K. pneumoniae* in Sri Lanka, and included mainly ST14 and ST147 (Hall et al., 2014). ST437 belongs to CC258 and is a single locus variant of the globally prevalent ST258, carrying *bla*_KPC_ in Brazil and *bla*_NDM_, *bla*_OXA-245_ (plasmid: IncL/M) in Spain (Seki et al., 2011; Oteo et al., 2013). *bla*_OXA–181_ was previously described in plasmid ColE, IncT and in the chromosome, which were all isolated from patients transferred from India, as well as described in plasmid IncX3 from China (Potron et al., 2011a; Dimou et al., 2012; Villa et al., 2013; Kayama et al., 2015; Liu et al., 2015; Partridge et al., 2015). ColE plasmid encoding various β-lactamase genes and *bla*_OXA–181_ is related to transposon Tn*2013*. Insertion sequence IS*Ecp1* was considered to be associated with *bla*_OXA–181_ acquisition, and its deletion may stabilize the resistance gene in the plasmid (Potron et al., 2013). *mobC* gene deletion in the strains may affect the frequency of conjugal plasmid mobilization (Zhang and Meyer, 1997).

In this study, all strains harbored quinolone-resistant determinants with quinolone resistance-determining region (QRDR) mutation on their chromosomes. A recent epidemiology study has shown the correlation of quinolone consumption and CRE in United States military health system, and another case-control study of CRE outbreaks in the Netherlands have determined quinolone use as the only risk factor for the acquisition of *bla*_OXA–48–like_ producing *Enterobacteriaceae* compared to other antibiotics use (Lesho et al., 2015; Dautzenberg et al., 2016). Possible mechanisms may be due to co-transfer of two plasmids bearing dual resistance mechanisms or recombination into one plasmid as described before (Rodríguez Martínez et al., 2014; Liu et al., 2015). Furthermore, the impact of different plasmids and fluoroquinolone resistant determinants on fitness cost has been found to vary with strains of different STs. This may have contributed to the spread of this particular clone (Tóth et al., 2014; Johnson et al., 2015). It was suggested that energetically favorable mutations with the double serine mutations in the QRDR region in the current study have been described to favor the fitness and dissemination of such clones (Fuzi et al., 2017). Although all strains were non-hypervirulent *K. pneumoniae* (negative for *rmpA*/*rmpA2* genes, non K1/K2 capsule serotypes), all strains encoded several yersiniabactin genes. These genes being on the integrative conjugative elements (ICE*Kp*) can contribute to the spread of *K. pneumoniae* in the population and may serve as a predictor of invasive infection (Lam et al., 2018).

In conclusion, we explored the genetic profile of multidrug resistant CRE in Sri Lanka. *bla*_OXA–181_ (through ColE-type plasmid) and yersiniabactin genes have disseminated to different STs of *K. pneumoniae*. We recommend active surveillance of high risk inpatients and long term studies to determine possible intra-unit transfer and facilitate infection control, especially as the length of stay in all instances where data was available is >72 h. Judicious antibiotics use especially that of quinolones is also recommended.

## Author Contributions

VP and ND conceptualized, collected, preliminarily analyzed, and approved the manuscript. MI, MH, and VL conceptualized, conducted, analyzed, drafted, and approved the manuscript. CZ, NL, KW, and CL contributed to sample storage and preparation, molecular analysis, and approval of the manuscript. CZ prepared the first draft of the manuscript.

## Conflict of Interest Statement

The authors declare that the research was conducted in the absence of any commercial or financial relationships that could be construed as a potential conflict of interest.
